# Case report: Cervical arterial dissections in the setting of recent COVID-19 infection

**DOI:** 10.3389/fstro.2024.1366947

**Published:** 2024-11-18

**Authors:** Sanghee Lim, Matthew M. Rode, Zafer Keser, Kelly D. Flemming

**Affiliations:** ^1^Department of Neurology, Mayo Clinic, Rochester, MN, United States; ^2^Mayo Clinic Alix School of Medicine, Mayo Clinic, Rochester, MN, United States

**Keywords:** arterial dissection, cervical arterial dissection, COVID-19, vasculitis, MR angiography

## Abstract

**Background:**

COVID-19 infections have been implicated in cerebral ischemia, but their relationship to cervical arterial dissections remains poorly characterized. Descriptions of cervical arterial dissections in patients with COVID-19 infections with details regarding their presenting symptomatology, imaging findings, and responses to treatment with antithrombotic therapy may be helpful to clinicians.

**Methods and observations:**

We present six adult cases of cervical arterial dissections in the setting of recent COVID-19 infections from 2021 to 2022 at our institution. Four cases presented with dissections involving the internal carotid artery, while two cases had dissections of bilateral vertebral arteries. In one patient, we found imaging evidence for a possible inflammatory process. All patients were treated with either antiplatelet agents or direct oral anticoagulants.

**Conclusions and relevance:**

COVID-19 infections may predispose patients to spontaneous cervical arterial dissections. Such patients can have variable neurologic presentations, though headaches and neck pain were common complaints. Most patients responded well to antithrombotic therapy, with improvement in symptoms and radiologic findings at follow-up. Clinicians should maintain a high degree of suspicion for cervical arterial dissections in patients who present acutely with severe headache/neck pain and/or new neurologic deficits in the setting of COVID-19 infections.

## Introduction

COVID-19 has been shown to be associated with an increased risk of stroke (Klok et al., 2020; Merkler et al., 2020; Oxley et al., 2020; Yaghi et al., 2020). Mechanisms of stroke in the context of COVID-19 are multifactorial and include hypercoagulability, endothelial injury, formation of anti-phospholipid antibodies, increased incidence of atrial fibrillation and hypokinetic heart conditions, and increased incidence of concurrent known stroke risk factors such as hypertension, diabetes, and coronary artery disease (Lindsberg and Grau, 2003; Merkler et al., 2020; Zhang et al., 2020; Rosenblatt et al., 2022). We report an emerging etiology of stroke in the context of COVID-19: cervical arterial dissections. Cervical artery dissections are responsible for 10–25% of strokes in young people under the age of 49 years (Putaala et al., 2009; Blum and Yaghi, 2015). Previous studies have reported a potential association between cervical artery dissections and concurrent respiratory and urinary infections, to date there have been at least 10 reports of cervical artery dissection in the context of COVID-19, yet many of these have poor clinical or radiographic follow up (Hernandez-Fernandez et al., 2020; Morassi et al., 2020; Patel et al., 2020; Gencler et al., 2021; Ghorbani et al., 2022; Mooney et al., 2022; Purdy et al., 2022; Sop and Allen, 2022; Spiewak et al., 2022). In this study, we share the experience of a single institution with a case series of cervical arterial dissections in COVID-19-positive patients and review the literature of the relationship between COVID-19 and cervical arterial dissection.

## Patient 1

A 41-year-old male presented to the emergency department 90 min after experiencing a unilateral temporal “thunderclap” headache with concurrent ipsilateral, painless monocular vision loss of his right eye. He had been having a cough for several days which he attributed to sinus problems but denied other symptoms concerning for upper respiratory tract infection. His past medical history was notable for type 1 diabetes mellitus on an insulin pump, hypertension, and hyperlipidemia. On physical exam, his blood pressure was 141/91. His right monocular visual field had improved to being able to detect motion by time of his evaluation in the ED. The remainder of the neurologic exam was unremarkable, including a fundoscopic exam and point-of-care tonometry. Laboratory studies, including ESR and CRP, were normal, except for COVID-19 positivity by PCR.

**Figure 1:**
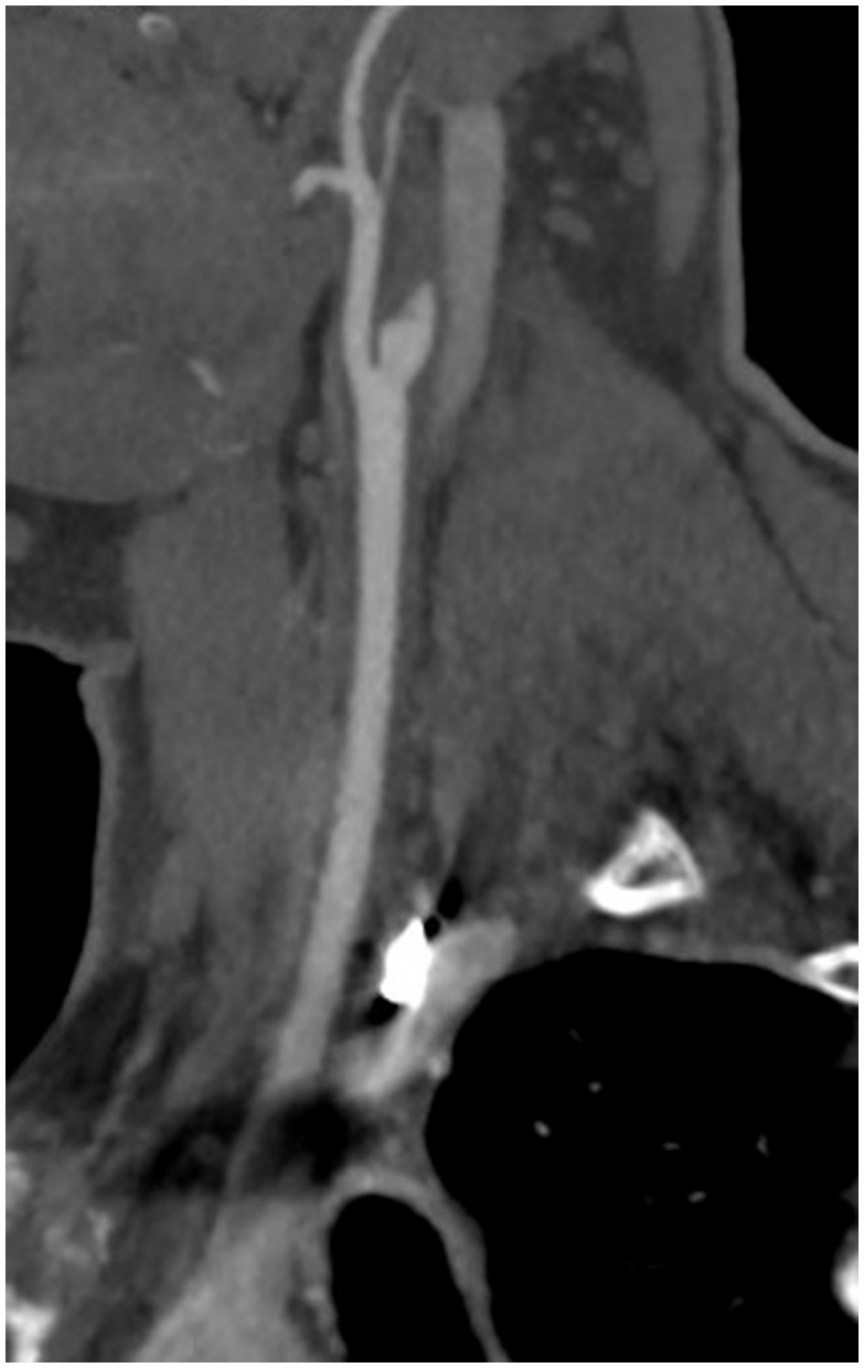
CT angiography demonstrating the “flame sign,” usually indicative of arterial dissection, in the right internal carotid artery.

A non-contrast enhanced CT scan of his head was negative for acute intracranial pathology, and he was not deemed a candidate for thrombolysis as more than 4.5 h had passed since symptom onset. CT angiography of his head and neck revealed dissection and complete occlusion of the right internal carotid artery (ICA) 1 cm distal to the ICA origin with reconstitution at the level of the supraclinoid segment (Figure 1). The anterior, middle, and posterior cerebral arteries were all patent, fed through the anterior communicating artery. Brain MRI showed no foci of diffusion restriction. MR-angiography confirmed the diagnosis of ICA dissection and occlusion without vasculitis. He denied a history of connective tissue disease, hypermobility, and head or neck trauma. The patient's vision had improved in the emergency department to baseline within 6 h. The patient was started on a heparin drip for 24 h and then transitioned to apixaban 5 mg twice daily. The patient was discharged without neurologic deficits. At 4 months, CT-angiogram demonstrated continued occlusion of the right ICA. Apixaban was transitioned to aspirin 325 mg daily. One year after discharge he had no deficits or signs of cerebral ischemia, and aspirin was decreased to 81 mg daily. Clinical and radiographic characteristics, treatment, and outcomes are reported for patients in this series as well as 13 previously reported cases in Table 1.

**Table 1:** Timeline of salient clinical encounters for all six cases, including prior to hospital admission, as well as post-admission follow-up visits.

**Case 1—Timeline**				
**Morning of admission**	**Hospital day #1**	**Hospital day #2**	**Hospital day #3**	**3 months post-discharge**
Acute onset unilateral thunderclap headache, right eye monocular vision loss	Mild improvement in right monocular vision deficits while in ED. Found to be COVID-19 positive on PCR. CTA notable for complete occlusion of right ICA with dissection. Started on heparin drip.	MRI without foci of diffusion restriction. Transitioned to apixaban 5 mg twice daily.	Discharged home without neurologic deficits.	CTA H&N demonstrating persistent total occlusion of right ICA with distal reconstitution to the level of ophthalmic artery via collateral flow.
**Case 2—Timeline**			
**8 days prior to initial presentation**	**Day of initial presentation**	**Hospital day #1**	**3 months post-discharge**
New onset fatigue, malaise, myalgia, pharyngitis with severe coughing fits. New neck pain.	Evaluated in the ED due to progressive neck pain; NIHSS 0. CTA H&N notable for bilateral ICA dissections	MRA head and neck notable for abnormal enhancement of bilateral ICAs. MRA chest/abdomen/pelvis negative for systemic vasculitides. Discharged home with 3 month course of aspirin and Plavix.	Repeat MRA head and neck notable for improved luminal flow and with mildly enlarged pseudoaneurysm.
**Case 3—Timeline**					
**2 days prior to initial presentation**	**Day of initial presentation**	**Hospital day #1**	**Hospital day #2**	**Hospital day #3**	**2 months post-discharge**
Subjective malaise, confusion at home. Left-sided hemibody weakness and left-sided facial droop noted by wife. Patient also reported headaches and transient right-sided visual changes.	Found to be COVID-19 PCR positive. CTA H&N demonstrated distal right ICA dissection with intramural hematoma and high-grade luminal narrowing.	Persistent headache, but with interval resolution of visual field deficits per patient.	MRI brain demonstrated multifocal embolic infarcts following a right MCA territory distribution. Aspirin added to antithrombotic regimen.	Discharged home in stable condition with exam notable only for mild satelliting of right upper extremity and subtle left-sided facial droop.	Improvement in ICA dissection noted on repeat CTA H&N with increased luminal size, though some aspects of repeat imaging concerning for possible fibromuscular dysplasia. Planned for follow-up imaging and clinic visit in 1 year. Neurologic exam stable. Aspirin stopped.
**Case 4—Timeline**						
**1 week prior to presentation**	**Day of presentation**	**Hospital day #1**	**2 weeks post-discharge**	**4 weeks post-discharge**	**3 months post-discharge**	**9 months post-discharge**
Development of upper respiratory tract infectious symptoms—sinus congestion, dysgeusia, anosmia, and coughing.	Minor motor vehicular accident while driving on snowy road due to “syncopal” event. Non-contrast enhanced head CT within normal limits. Admitted for observation. COVID-19 PCR positive.	Discharged home in stable condition. Arranged for local follow-up with Neurology and Family Medicine.	Recommended for MRA of head and neck by Neurology after outpatient clinic visit.	MRA head and neck notable for left ICA dissection with intramural hematoma formation. Started on apixaban 5 mg twice daily.	Seen by both Neurosurgery and Vascular Neurology in outpatient setting. Stable neurologic exam. Repeat MRA head and neck with stable luminal narrowing of left ICA. Transitioned from apixaban to aspirin 81 mg daily.	Stable neurologic exam without deficits. Scheduled for follow-up in 1 year's time.
**Case 5—Timeline**						
**2 weeks prior to admission**	**Morning of admission**	**In the emergency department**	**Angiography suite**	**Neurosciences intensive care unit**	**Stroke floor course**	**1 month post-discharge**
COVID-19 infection. Tested positive on home antigen kit, with symptoms of pharyngitis and headaches.	Visited local chiropractor due to ongoing headaches. Developed acute onset nausea, vertigo, and dysarthria after neck manipulation.	NIHSS of 4 on initial assessment, with worsening exam while in CT scanner with bilateral upper extremity weakness and drift. Received IV tenecteplase.	Total occlusion of left vertebral artery noted with severe stenosis of right vertebral artery. Two stents placed in right vertebral artery with improvement in neurologic symptoms.	Follow-up CT scan demonstrated intraparenchymal hemorrhage of right occipital lobe. Repeat imaging 24 h after demonstrated ongoing stability.	Transferred to inpatient Stroke floor on hospital day #2. Started on dual antiplatelet therapy (DAPT) with aspirin 81 mg daily and ticagrelor 90 mg twice daily. Discharged home in stable condition on hospital day #4.	Total recanalization of left vertebral artery noted on repeat CTA H&N. Stable neurologic exam without deficits. Recommended to continue DAPT for additional 2 months.
**Case 6—Timeline**						
**2 weeks prior to admission**	**Hospital day #1**	**Neurosciences ICU course (hospital days #1–#6)**	**Hospital day #7–#8**	**Hospital days #9–#28**
COVID-19 infection with symptoms of pharyngitis, headaches, high fevers, and recurrent coughing bouts.	Brought into ED due to lethargy and vomiting. CTA H&N demonstrated bilateral vertebral artery dissections with intraluminal thrombus in right vertebral artery. MRI brain demonstrated right cerebellar infarct. Admitted to the Neurosciences ICU. Started on aspirin 325 mg daily.	Developed effacement of 4th ventricle requiring Mannitol. Neurologic exam improved with imaging stability. Transferred to stroke floor.	Shortly after transfer to floor, pt performed a typical “neck cracking” motor tic (noted history of this as part of his autism spectrum disorder) and developed acute onset left gaze deviation and left sided weakness. CTA H&N demonstrated propagation of clot into the basilar artery. New infarcts noted in the pons and inferior cerebellum.	Briefly transferred to Neurosciences ICU for monitoring in setting of new strokes. Transferred back to stroke floor on hospital day #10. Started on heparin drip, and transitioned to apixaban 5 mg twice daily. Discharged to inpatient rehabilitation on hospital day #28.

ED, Emergency Department; NIHSS, National Institutes of Health Stroke Scale score; DAPT, Dual Antiplatelet Therapy; ICA, Internal Carotid Artery; PCR, Polymerase Chain Reaction; CTA H&N, Computed Tomography Angiogram of the Head and Neck; MRI, Magnetic Resonance Imaging; MRA, Magnetic Resonance Angiogram; ICU, Intensive Care Unit; COVID-19, Coronavirus Disease 2019.

## Patient 2

A 42-year-old male with no significant past medical history developed symptoms of fatigue, malaise, myalgias, pharyngitis, and cough. Coughing fits were prolonged at times, with the patient reporting he would sometimes “turn blue.” He was diagnosed with COVID-19 and treated himself symptomatically. He also experienced neck pain during this initial illness which he initially attributed to generalized myalgia. However, over time his infectious symptoms improved, but his neck pain progressed. Eight days after initial symptom onset, he presented to the emergency department with left occipital head and anterior left neck pain. He had no other neurologic symptoms or signs on exam with a National Institute of Health Stroke Scale score of 0.

**Figure 2:**
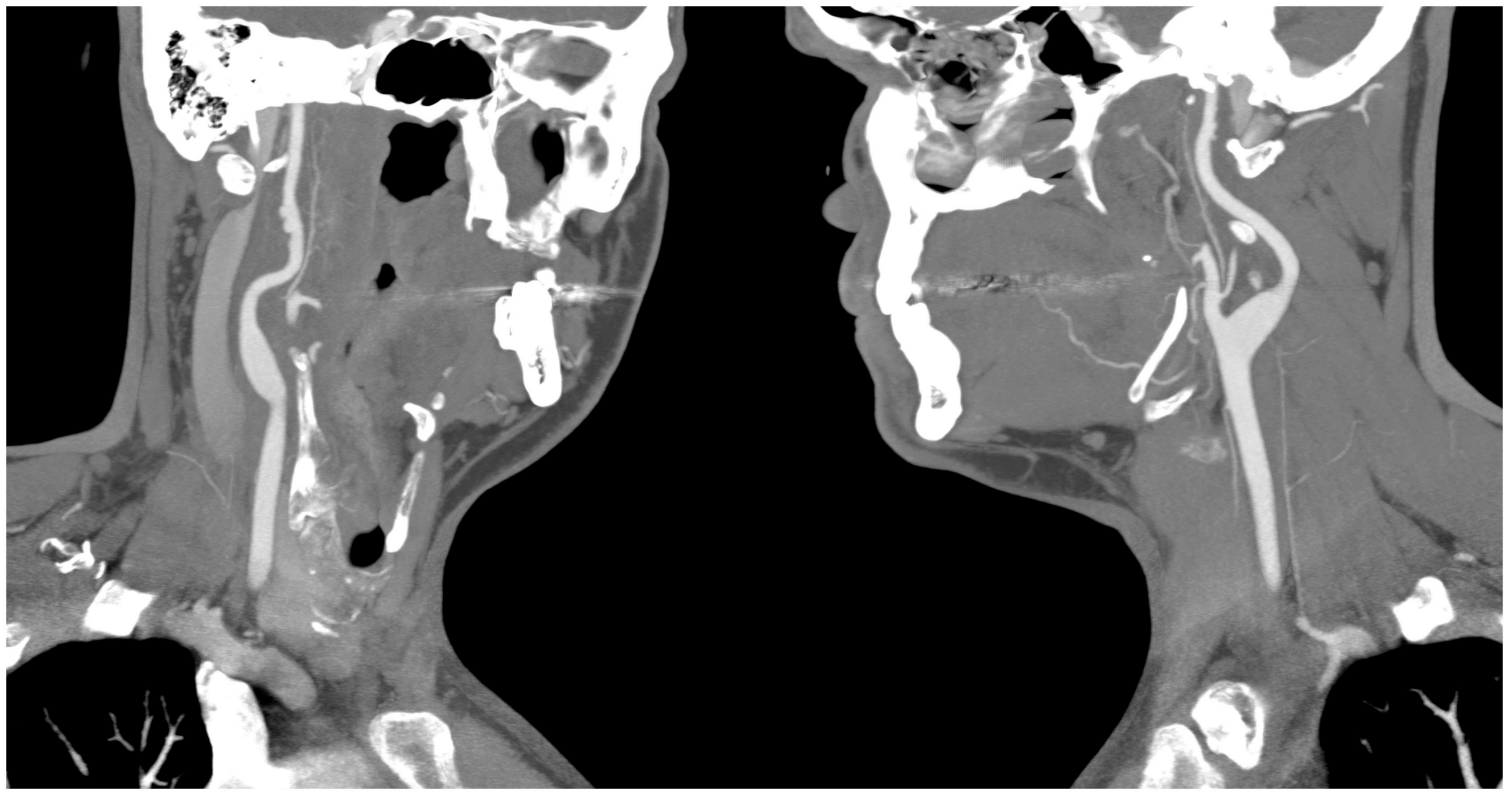
CT angiography showing bilateral pseudoaneurysms with the **(left panel)** showing the patient's right internal carotid artery, and the **(right panel)** showing the patients left internal carotid artery.

**Figure 3:**
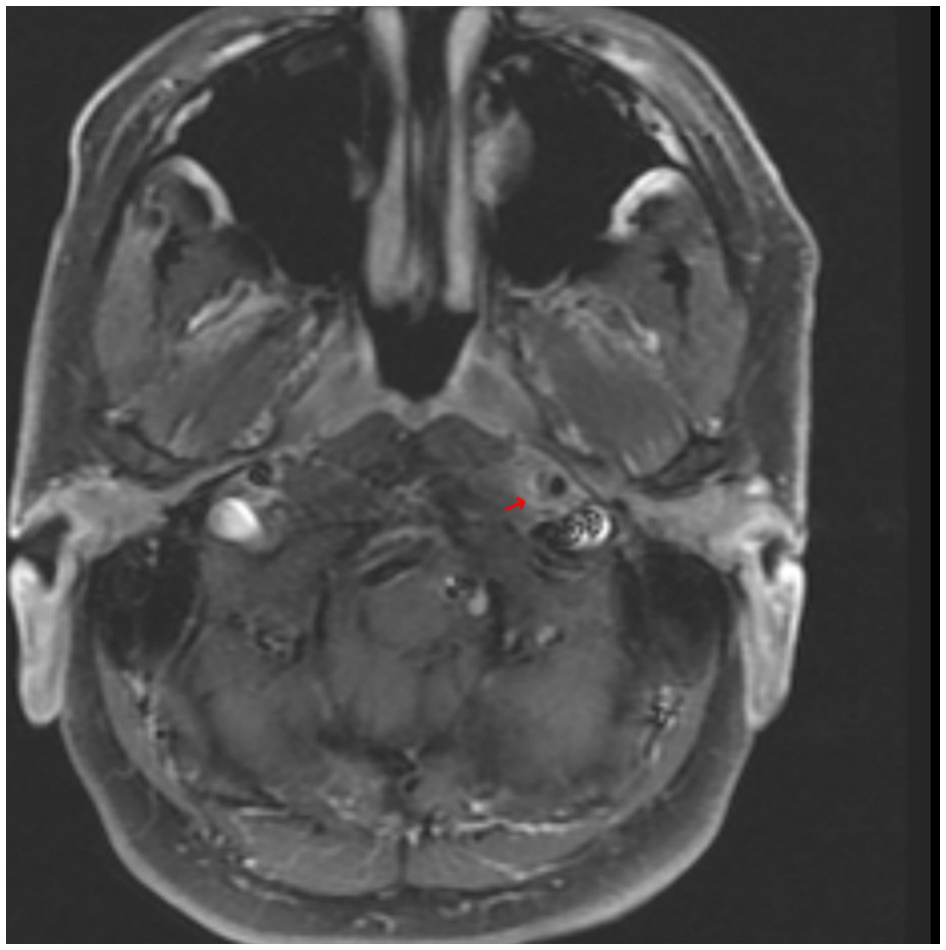
An axial T1-weighted MR angiography post-gadolinium image demonstrating hyperintensity surrounding the lower left ICA near the bifurcation and corresponding to the internal carotid artery wall; arrow indicates area of suspected perivascular enhancement in the region of the dissection in the distal cervical segment.

CT angiogram of the head and neck showed dissections of bilateral extracranial internal carotid arteries with mild luminal narrowing and associated small pseudoaneurysms (Figure 2). MR angiogram confirmed dissections with intramural hematomas noted on T1 fat saturated sequencing, but also noted abnormal enhancement of the extracranial right internal carotid artery and left internal carotid artery near the bifurcation surrounding the dissection (Figure 3), concerning for an inflammatory process. Laboratory studies, including a complete blood count, erythrocyte sedimentation rate, and c-reactive protein, were within normal limits. MR angiogram of the chest, abdomen, and pelvis demonstrated no additional signs of visceral vasculitis. The patient was treated with aspirin and clopidogrel for 3 months. At 3-month follow up, repeat MR angiogram demonstrated improved luminal flow, but slightly enlarged pseudoaneurysm.

## Patient 3

**Figure 4:**
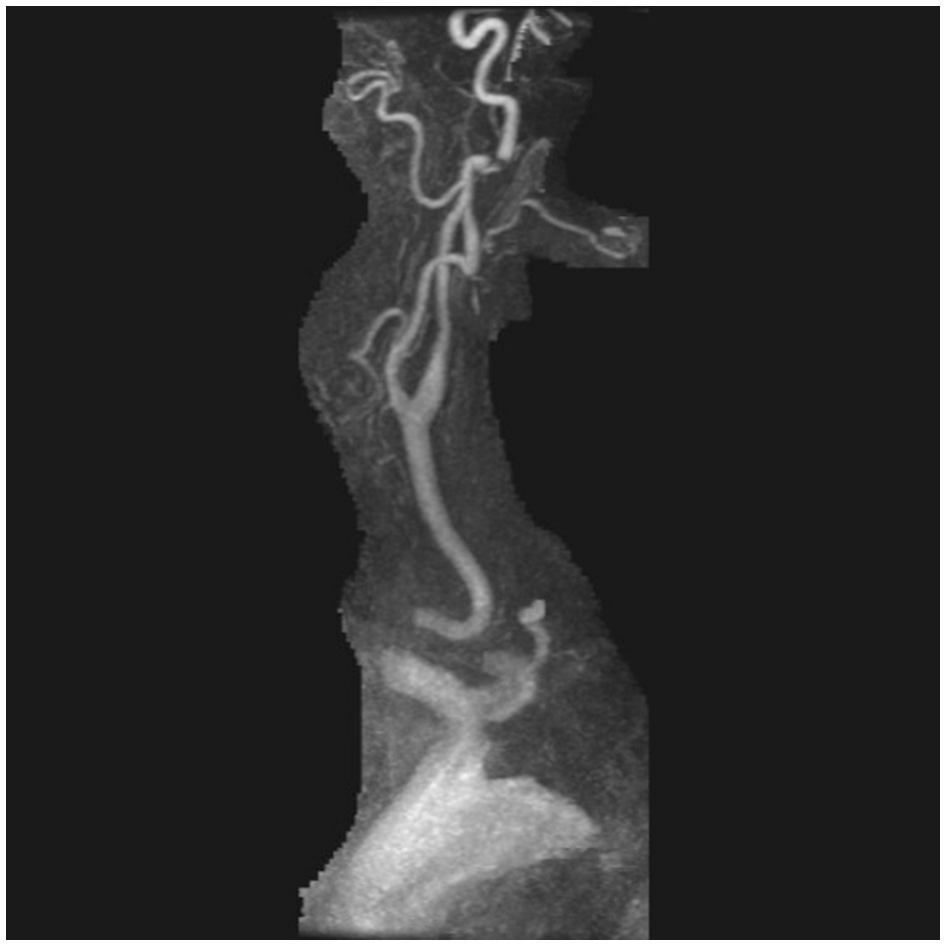
MR angiography post-gadolinium reconstruction of the right internal carotid artery demonstrating disruption of flow by a dissecting pseudoaneurysm.

A 74-year-old male with a past medical history notable for atrial fibrillation on apixaban and prior cardioversion presented to a local Emergency Department with a 2-day history of subjective malaise and mild confusion, including difficulty with previously familiar activities, such as using a routine computer program. On the day of hospital presentation, his wife also reported that the patient had been experiencing weakness of his left hemibody in addition to a left-sided facial droop; the patient additionally endorsed a headache and right-sided visual changes that were transient. He denied all other constitutional symptoms, including fevers, cough, or myalgias. On laboratory work-up, he was found to be COVID-19 positive, and imaging with CT and MR angiograms of his head and neck showed a dissection of the distal right internal carotid artery and a pseudoaneurysm associated with high-grade luminal narrowing (Figure 4). MR imaging of his brain confirmed the presence of multifocal embolic infarcts following a right MCA (Middle Cerebral Artery) distribution. His admission neurologic exam was notable for left facial droop and subtle satelliting of his right upper extremity around the left upper extremity, indicating left-sided weakness. The patient was continued on his anticoagulation therapy with apixaban, and daily aspirin 81 mg was added to his antithrombotic regimen. At 3-month follow-up, repeat CT angiography of his head and neck demonstrated a small pseudoaneurysm of the right distal ICA without stenosis, as well as evidence of possible fibromuscular dysplasia in the distal ICAs bilaterally; reassuringly, his repeat neurologic exam was without overt deficits. Aspirin was thus discontinued, but the patient remained on apixaban for stroke prevention due to atrial fibrillation. Two years after dissection, the patient has had no new vascular neurologic events.

## Patient 4

A 44-year-old male with past medical history significant for hypertension was brought to his local Emergency Department after a syncopal episode while driving his car at 20 miles per hour, resulting in a mild whiplash injury to his neck. His admission physical exam was unremarkable, without overt signs of trauma. He was subsequently admitted to the hospital's observation unit. He underwent laboratory investigations that included a complete blood count, basic metabolic panel, and screen for drugs of abuse, which was only notable for a mild thrombocytopenia at 106,000 platelets per microliter of blood, and decreased absolute lymphocyte count at 450 lymphocytes per microliter of blood. He was also found to be COVID-19 positive by PCR. The patient reported that he had been mildly symptomatic from this COVID-19 infection with sinus congestion and intermittent coughs, as well as anosmia and dysgeusia for ~1 week prior to his ED presentation. He underwent a non-contrast enhanced CT scan of his head, which was negative for acute intracranial pathology. He was admitted to the hospital for monitoring, and after one night of observation to ensure clinical stability, the patient was discharged with scheduled outpatient follow-up visits with Family Medicine and Neurology.

**Figure 5:**
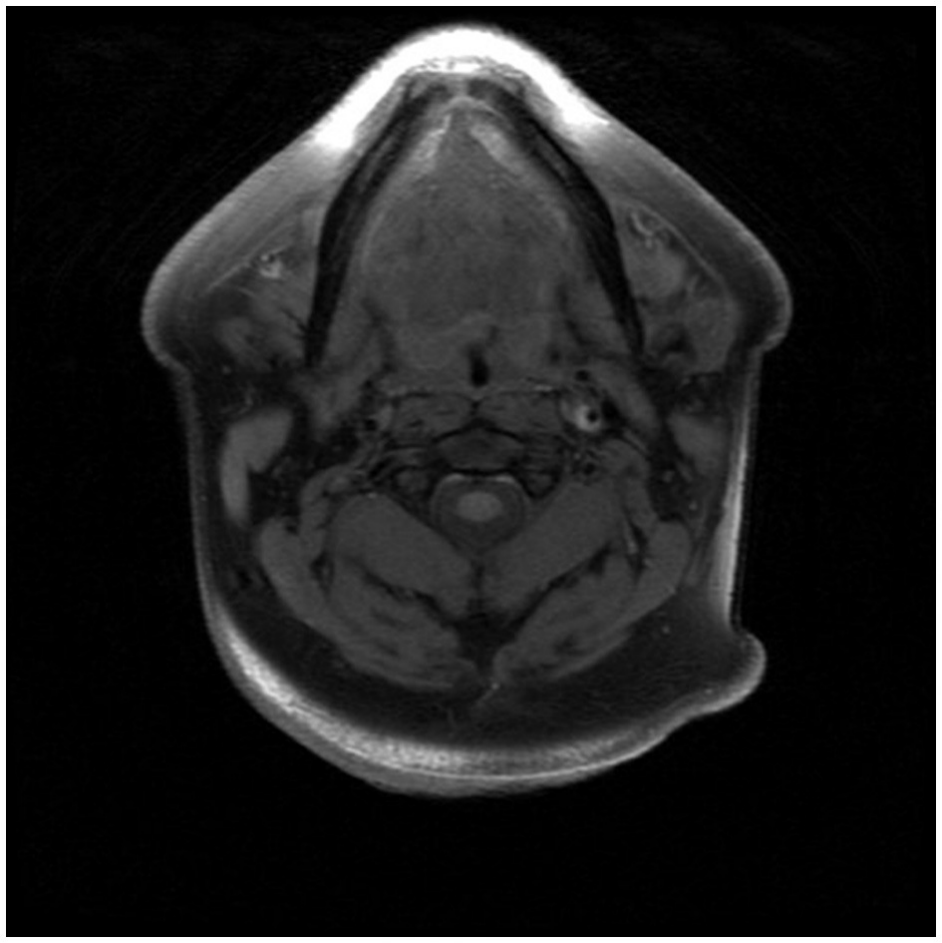
MR angiography T1 weighted, pre-gadolinium image demonstrating hyperintensities surrounding the left internal carotid artery, indicative of an intraluminal hematoma.

His outpatient neurologist recommended further imaging studies, and a subsequent MR angiogram of his head and neck was performed. This demonstrated crescentic wall thickening and luminal narrowing of the left cervical ICA with intramural hematoma formation (Figure 5). He was treated with apixaban 5 mg twice daily and was recommended to obtain repeat vessel imaging of his head and neck after 3 months. He was found to be clinically stable without neurologic deficits at this 3-month follow-up visit, and received a repeat MR angiogram of his neck, which showed improvement of his left ICA dissection. He was subsequently transitioned to aspirin 81 mg daily and was found to be without any new symptoms at a subsequent 2 year follow-up visit with vascular neurology. The patient has had no recurrent syncope with an unrevealing cardiac work up. The syncope was attributed to COVID-19 infection. It was believed that the accident triggered the carotid dissection; although, the temporal relationship of the syncope and dissection cannot be confirmed.

## Patient 5

A 30-year-old female without significant prior medical history was brought by ambulance to our Emergency Department after an episode of acute onset disorientation, vertigo, nausea, and dysarthria during a visit to her chiropractor. She had experienced symptoms of pharyngitis and headaches with a recent COVID-19 infection ~2 weeks prior to her hospital presentation. As such, she sought out the care of a local chiropractor in hopes of remedying her headaches which had persisted. After the first few movements of neck manipulation, she experienced new onset nausea and vertigo with some difficulty speaking. When she brought these symptoms up to her chiropractor, her neck was reportedly counter-maneuvered in the opposite direction, with a further worsening of symptoms, now with noted pooling of oral secretions, inability to swallow, and rapidly increasing severity of her headache. At this point, an ambulance was called. On initial assessment, her National Institute of Health Stroke Scale was 4 for right-sided hemiataxia and dysarthria.

**Figure 6:**
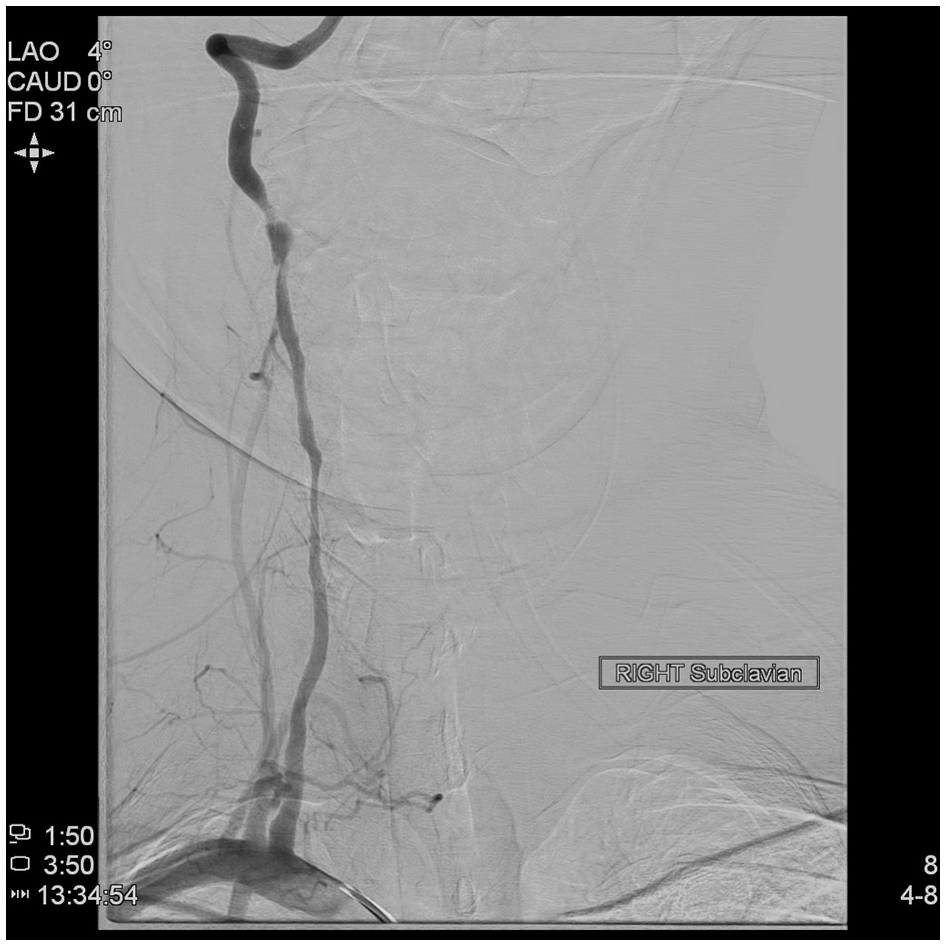
Direct angiography of the right subclavian artery demonstrating severe stenosis and pseudoaneurysm of the right vertebral artery.

An acute Code Stroke was called; her clinical presentation continued to worsen, with new onset of bilateral upper extremity weakness and drift while in the CT scanner, and she received IV Tenecteplase. CT angiography of her head and neck showed bilateral vertebral artery dissections with occlusion of the left V3 segment and narrowing of the left intradural vertebral artery concerning for intra-cranial extension of the dissection flap. She was urgently taken to the angiography suite, where a total occlusion of the left vertebral artery with severe stenosis and pseudoaneurysm of the right vertebral artery were found (Figure 6). Two stents were placed in both proximal and distal segments of the right vertebral artery with significant improvement in neurologic symptoms. She was thereafter taken to the Neurosciences Intensive Care Unit, where a follow-up non-contrast enhanced CT scan of her head demonstrated a new intraparenchymal hemorrhage involving the right occipital lobe. Repeat CT imaging of her head demonstrated stability of this intraparenchymal hemorrhage. She was started on dual-antiplatelet therapy with aspirin 81 mg daily and ticagrelor 90 mg twice daily after ensuring ongoing clinical stability. On her 1-month follow-up visit with Vascular Neurology, repeat CT angiogram of her head and neck demonstrated total recanalization of her left vertebral artery and partial recanalization of the right vertebral artery with patent stents, and her neurologic exam was without any deficits. At 6 months, CT angiography demonstrated resolution of the right pseudoaneurysm as well as stable luminal patency. She was asymptomatic; therefore, ticagrelor was discontinued with plans to continue aspirin for life.

## Patient 6

**Figure 7:**
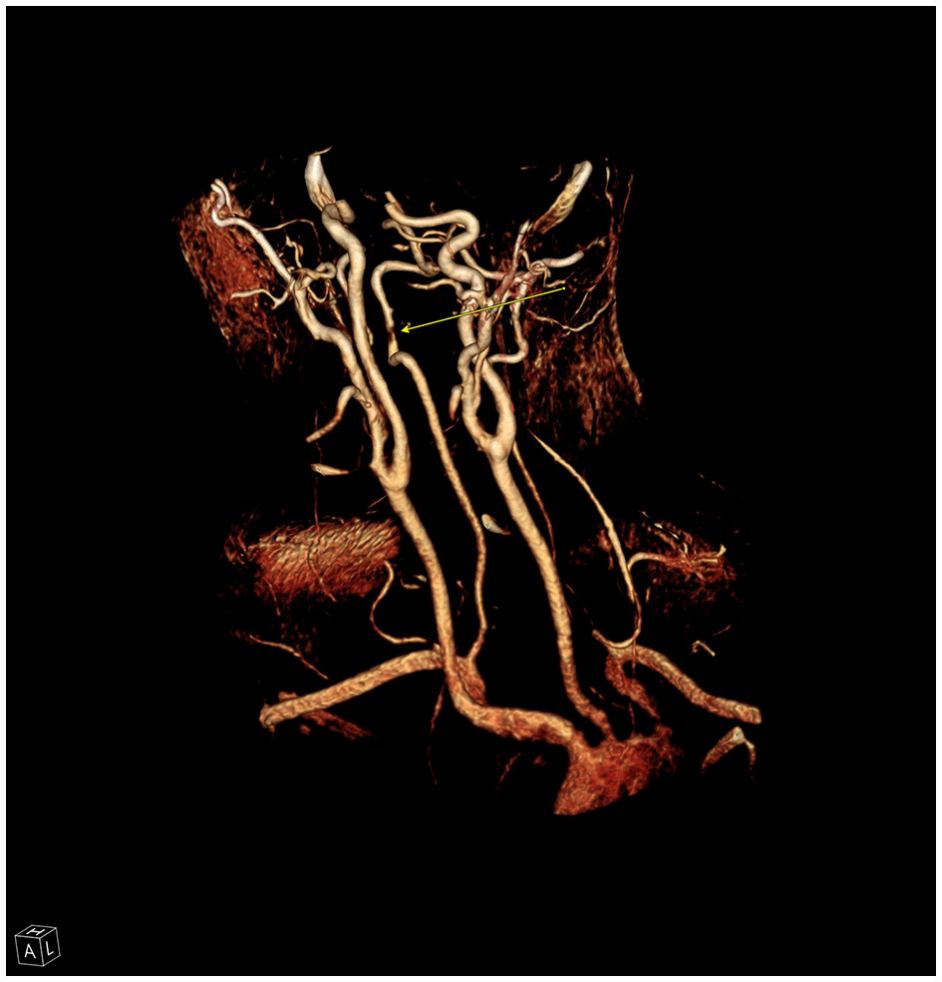
Reconstruction from CT angiography of the neck demonstrating high grade stenosis (arrow) of the right vertebral artery due to dissection and intraluminal thrombus.

A 29-year-old male with past medical history notable for autism spectrum disorder, attention deficit hyperactivity disorder on lisdexamfetamine, and a recent COVID infection ~2 weeks prior presented to our Emergency Department with acute onset vomiting, headaches, dizziness, and lethargy. Per his family, the patient had had significant symptoms from his COVID-19 infection, including bouts of coughing fits and high fevers. Of note, his autism spectrum disorder was also associated with recurrent motor tics to “crack” his neck, characterized by volitional hyper-extension of his neck bilaterally. Diagnostic imaging with CT angiography of his head and neck showed bilateral vertebral artery dissections with an intraluminal thrombus in the right vertebral artery (Figure 7) and MRI brain demonstrated a large right cerebellar infarct following both Superior Cerebellar Artery and Posterior Inferior Cerebellar Artery distributions. He was admitted to the Neurosciences Intensive Care Unit (NSICU) and was started on aspirin 325 mg daily due to concern for being at high risk for hemorrhagic conversion. The patient developed cerebellar edema with effacement of the fourth ventricle on surveillance imaging while in the NSICU, and required mannitol for management of this edema. His presentation initially continued to improve along with partial resolution of intraluminal thrombus in the right vertebral artery, and he was transferred to the stroke service on hospital day #8.

Unfortunately, on hospital day #9, the patient performed a sudden forceful movement of his neck while working with physical therapy, and developed acute onset left-sided gaze deviation and left-sided weakness. An acute Code Stroke was called, and repeat imaging with CT angiogram showed propagation of the right vertebral artery intraluminal thrombus into the basilar artery, with MR imaging demonstrating new acute infarcts in the right mid and inferior pons, as well as the inferior cerebellar vermis. Although endovascular therapy was considered, given the size of the existing strokes and partial occlusion of the basilar artery, maximal medical therapy with low-rate heparin drip was initiated instead. His neurologic exam was notable for left-sided hemiparesis, right-sided palsies of cranial nerves 6 and 7, dysarthria and dysphagia. On hospital day #28, he was discharged to acute inpatient rehabilitation for ongoing recovery. He was discharged home with family help after an additional 21 days of rehabilitation. MR angiography 4 months after initial diagnosis demonstrated resolution of the narrowing and irregularity of the vertebral arteries with normal vascular patency. After this, apixaban was transitioned to aspirin 81 mg daily. Two years after dissection he continues to struggle with left spastic hemiparesis.

## Discussion

We report on six cases of cervical artery dissection in the context of COVID-19 infection from a single institution between 2021 and 2022. Four cases presented with dissections involving the internal carotid artery, while two cases had dissections of bilateral vertebral arteries. Intraluminal hematoma was detected in two patients, and three patients presented with pseudoaneurysms. In one patient, we found imaging evidence for a possible inflammatory process. All patients were treated with either antiplatelet agents or direct oral anticoagulants. Clinical outcomes were reassuring in five of the cases, while one patient suffered from persistent symptoms of a resulting ischemic stroke (without recurrent stroke or dissection).

Previous cases of adults with cervical artery dissection in the context of COVID-19 reported cough somewhat more frequently (three of five cases reporting on this symptom; Ghorbani et al., 2022; Purdy et al., 2022; Spiewak et al., 2022) than in our series, where only two of six patients reported cough. Five of the 10 previously reported dissections in adults occurred in the posterior circulation (Hernandez-Fernandez et al., 2020; Morassi et al., 2020; Patel et al., 2020; Mooney et al., 2022; Purdy et al., 2022) while four of our six cases were in the carotid arteries. Ischemic stroke occurred in 6/10 previous adult cases (Hernandez-Fernandez et al., 2020; Mooney et al., 2022; Purdy et al., 2022; Sop and Allen, 2022; Spiewak et al., 2022), Of note, there has also been at least three reports of subarachnoid hemorrhage due to dissecting intracranial aneurysms in the context of COVID-19 (Al Saiegh et al., 2020; Dakay et al., 2020; Sato et al., 2022).

Trager et al. (2023) performed a retrospective matched cohort study of adults with cervical artery dissection who had undergone COVID-19 testing. The incidence of cervical artery dissection was similar between patients with COVID-19 (0.0045%) and those who tested negative for COVID-19 (0.0041%; Trager et al., 2023). Wahood et al. found that hospitalized patients with cervical artery dissection and COVID-19 had similar rates of home discharge and mortality when controlling for known confounders (Wahood et al., 2024). Overall, there is no evidence that COVID-19 infection independently increases risk of cervical artery dissection; although, providers should maintain awareness of known risk factors such as cough (Micheli et al., 2010) in the context of COVID-19.

Research efforts in the setting of rapid clinical advancements in the treatment of COVID-19 have implicated multiple pro-inflammatory signaling cascades, especially those involving IL-6, in the pathogenesis of cytokine release storms with resultant hyperactive host immune response resulting in multi-organ damage (Ragab et al., 2020; Wang and Perlman, 2022). This has resulted in the trialing of several agents for the management of such an inflammatory state, including small molecule inhibitors either directly targeting IL-6 or those impinging on downstream signaling axes, such as Tocilizumab, Baricitinib, and Tofacitinib (Chen et al., 2021; Wang and Perlman, 2022). Interestingly, IL-6 and related signaling pathways have been significantly implicated in the pathogenesis of arterial dissections, in both research models and patients, vis-à-vis activation of matrix metalloproteinases, immune cell recruitment, and ultimately, vessel medial wall degeneration (Grond-Ginsbach et al., 2010; Ju et al., 2014). As such, it is tempting to postulate that this pro-inflammatory signalome activation secondary to COVID-19 infection contributed to the pathogenesis of cervical artery dissections in our patients who were otherwise mostly young and without uncontrolled or overt cardio- and cerebrovascular risk factors. This is particularly the case for our second patient, who demonstrated neuro-imaging evidence consistent with a possible inflammatory process, noting that vasculitis is a known complication of COVID-19 infection, including those involving the central nervous system (Patel et al., 2020; Timmons et al., 2021). It is also possible that mechanical factors could have played a role, given significant reports of recurrent coughing fits secondary to COVID-19 infection in two of our patients, as well as history of neck manipulation, either volitional or non-volitional, in three patients (Micheli et al., 2010). However, such exposures to minor trauma are generally not associated with increased risk for cervical arterial dissections, save for in a minority of patients with defined connective tissue disorders, such as Marfan Syndrome or Ehlers-Danlos Syndrome (Giossi et al., 2014). Infections represent an additional risk factor for cervical arterial dissections and viral infections are thought in particular to increase risk for cervical arterial dissections by way of promoting focal arteriopathy (Debette and Leys, 2009). Interestingly, recent analyses of large public health databases have implicated COVID-19 with increased rates of pediatric arterial ischemic strokes secondary to focal cerebral arteriopathy (Tudorache et al., 2024). Endothelial cell dysfunction is a major hallmark of COVID-19 infections, and this has been attributed on the molecular level to the affinity of the viral spike protein for SARS-CoV2 to the ACE2 receptor, which is notably expressed extensively on endothelial and epithelial cells (Jin et al., 2020; Otifi and Adiga, 2022). Post-mortem autopsy-based studies of COVID-19 patients have implicated endothelium dysfunction as being a major pathophysiologic mechanism underlying pro-thrombotic phenomena as well as cardiac and renal dysfunction seen in severe COVID-19 infections (Varga et al., 2020). *In vitro* studies have demonstrated that COVID-19 infection contributes in a wide variety of mechanisms to disrupt not only the endothelial layer, but the pan-vasculature surrounding site of initial viral injury, including through dysregulation of local nitric oxide release, activation of oxidative stress responses, and induction of leukocyte activation through multiple pro-inflammatory signals (Xu et al., 2023). As such, given recent population-based studies demonstrating increased risk for focal arteriopathy with COVID-19 infections, as well as robust experimental data supporting the ability of SARS-CoV2 to promote both localized and generalized pan-vascular injury, a plausible mechanism by which infectious illness with COVID-19 promotes increased risk for cervical arterial dissections could be postulated as follows. Minor trauma, such as neck manipulation or whiplash injury, likely contribute to the underlying pathology by creating small tears in the tunica intima that would otherwise have been asymptomatic; however, in the pro-inflammatory milieu and arteriopathic background generated by an ongoing COVID-19 infection, these tears subsequently propagate and create larger arterial dissections (Micheli et al., 2010). Further studies are certainly warranted to better establish the relative contributions of each risk factor to their impact on incidence of cervical arterial dissections.

## Conclusion

We describe a series of six patients with cervical artery dissections in the context of COVID-19. Possible mechanisms by which COVID-19 infections increase risk for such dissections include increased inflammatory signaling, mechanical factors, and endothelium dysfunction. Providers should maintain awareness of the relationship between COVID-19 and cough, a known risk factor for cervical artery dissection.

## Data Availability

The datasets presented in this article are not readily available because of ethical and privacy restrictions. Requests to access the datasets should be directed to the corresponding author.
